# Predictive modelling of linear growth faltering among pediatric patients with Diarrhea in Rural Western Kenya: an explainable machine learning approach

**DOI:** 10.1186/s12911-024-02779-7

**Published:** 2024-12-02

**Authors:** Billy Ogwel, Vincent H. Mzazi, Alex O. Awuor, Caleb Okonji, Raphael O. Anyango, Caren Oreso, John B. Ochieng, Stephen Munga, Dilruba Nasrin, Kirkby D. Tickell, Patricia B. Pavlinac, Karen L. Kotloff, Richard Omore

**Affiliations:** 1 Kenya Medical Research Institute- Center for Global Health Research (KEMRI-CGHR), P.O Box 1578-40100, Kisumu, Kenya; 2 Department of Information Systems, University of South Africa, Pretoria, South Africa; 3 Department of Medicine, Center for Vaccine Development and Global Health, University of Maryland School of Medicine, Baltimore, MD USA; 4 Department of Global Health, University of Washington, Seattle, USA

**Keywords:** Machine learning, Linear growth faltering, Pediatric, Diarrhea, Prediction

## Abstract

**Introduction:**

Stunting affects one-fifth of children globally with diarrhea accounting for an estimated 13.5% of stunting. Identifying risk factors for its precursor, linear growth faltering (LGF), is critical to designing interventions. Moreover, developing new predictive models for LGF using more recent data offers opportunity to enhance model accuracy, interpretability and capture new insights. We employed machine learning (ML) to derive and validate a predictive model for LGF among children enrolled with diarrhea in the Vaccine Impact on Diarrhea in Africa (VIDA) study and the Enterics for Global Heath (EFGH) ― Shigella study in rural western Kenya.

**Methods:**

We used 7 diverse ML algorithms to retrospectively build prognostic models for the prediction of LGF (≥ 0.5 decrease in height/length for age z-score [HAZ]) among children 6–35 months. We used de-identified data from the VIDA study (*n* = 1,106) combined with synthetic data (*n* = 8,894) in model development, which entailed split-sampling and K-fold cross-validation with over-sampling technique, and data from EFGH-Shigella study (*n* = 655) for temporal validation. Potential predictors (*n* = 65) included demographic, household-level characteristics, illness history, anthropometric and clinical data were identified using boruta feature selection with an explanatory model analysis used to enhance interpretability.

**Results:**

The prevalence of LGF in the development and temporal validation cohorts was 187 (16.9%) and 147 (22.4%), respectively. Feature selection identified the following 6 variables used in model development, ranked by importance: age (16.6%), temperature (6.0%), respiratory rate (4.1%), SAM (3.4%), rotavirus vaccination (3.3%), and skin turgor (2.1%). While all models showed good prediction capability, the gradient boosting model achieved the best performance (area under the curve % [95% Confidence Interval]: 83.5 [81.6–85.4] and 65.6 [60.8–70.4]) on the development and temporal validation datasets, respectively.

**Conclusion:**

Our findings accentuate the enduring relevance of established predictors of LGF whilst demonstrating the practical utility of ML algorithms for rapid identification of at-risk children.

**Supplementary Information:**

The online version contains supplementary material available at 10.1186/s12911-024-02779-7.

## Introduction

Diarrhea, a global public health problem with greatest burden in low- and middle-income countries (LMICs) [1], is a leading etiology of malnutrition among children in LMICs, in part due to anorexia, decreased absorptive function, mucosal damage, catabolism and nutrient exhaustion [1, 2]. It has been reported that the cumulative burden of diarrhea days directly correlates with the degree of nutritional failure among children during early childhood and that catch-up growth does not appear to make up for the deficit [3]. Linear growth faltering (LGF), a precursor to stunting (height-for-age z-score [HAZ] <−2), is one form of malnutrition that results from protracted nutritional deprivation [4]. Stunting affects one-fifth of children globally and one-third of children in LMICs [5]. Globally, 13.5% of stunting cases are attributed to diarrhea [6]. Additionally, a vicious cycle of diarrhea and malnutrition can occur as malnutrition weakens the body’s defense against future diarrheal episodes resulting in more frequent and longer diarrheal illnesses. While LGF is a precursor to stunting, it also independently affects child development and health outcomes, such as cognitive delays, increased susceptibility to infections, and the risk of relapse into wasting after recovery [7, 8]. Furthermore, the effects of stunting can be severe and protracted, with reduced cognitive development, persistent poor health, and elevated risk of mortality [9]. Long term complications can include an increased risk of cardiovascular disease, type 2 diabetes, and obesity in adulthood [10, 11].

The timely and accurate identification of children at increased risk of LGF is crucial for early nutritional and healthcare interventions as well as efficient allocation of public health resources, efforts that could help to avert the associated negative outcomes. Data-driven predictive models could be leveraged to this end and a number of research efforts exist in the prediction of LGF among children with diarrhea [12, 13]. These studies utilized clinical and sociodemographic data from the Global Enteric Multicenter Study (GEMS), conducted between 2007 and 2011, to develop predictive models. The models lacked explainable methodologies to improve interpretability and demonstrated moderate discrimination, with areas under the ROC curve (AUC) of 67.0% for Branders et al. [12] and 75.0% for Ahmed et al. [13], respectively. While the existing models provide a valuable starting point, shifts in the study population over time may affect the predictive performance of these models [14, 15]. Moreover, development of new models using more recent and pertinent data offers the opportunity to improve model accuracy, enhance interpretability of models and capture new perspectives and insights into this public health problem. We used machine learning (ML), which has been adopted in public health and clinical practice to rapidly develop data-driven clinical prediction models, to develop and temporally validate predictive models for LGF among children aged < 5 years with diarrhea in rural Western Kenya.

## Methods

### Data sources

This retrospective study used data collected from the Kenyan site (in Siaya County) of two related diarrheal studies: The Vaccine Impact on Diarrhea in Africa (VIDA) study [16] for model development and evaluation; and the Enteric for Global Health (EFGH) *Shigella* surveillance study [17, 18] for temporal validation.

### Development cohort

VIDA was designed to assess the population-based incidence, etiologies, and adverse clinical consequences of diarrhea following rotavirus vaccine introduction in children aged 0–59 months residing in censused populations in 3 African countries. Moderate-to-severe diarrhea (MSD) cases, defined as children in 3 age strata (0–11, 12–23, and 24–59 months) presenting with diarrhea (defined as ≥ 3 looser-than-normal stools within 24 h) that began within the past 7 days after ≥ 7 diarrhea-free days and had ≥ 1 of the following: sunken eyes, poor skin turgor, dysentery, intravenous rehydration, or required hospitalization, were enrolled from sentinel health centers (SHCs) serving the health and demographic surveillance systems population at each site. The aim was to enroll 8–9 MSD cases in each age stratum per fortnight. 1–3 diarrhea-free controls matched by age, gender and geographical location were enrolled within 14 days of case enrolment. Follow-ups were conducted between 49 and 91 days after enrolment. We utilized data collected from cases enrolled at the VIDA Kenya site over a 36 months period from May 2015 and July 2018 restricting to children aged 6–35 months to make the development and temporal validation cohorts comparable. The study design, clinical and epidemiological methods for VIDA have been described elsewhere [16, 19].

In addition to the VIDA data (*n* = 1,106), we generated a synthetic dataset (*n* = 8,894) based on the VIDA data using the synthpop package [20] to increase the sample size and to enable the algorithms to generate more stable and reliable predictions that are less sensitive to noise in the data. The variables of the synthetic dataset were compared to the original training dataset with the synthetic dataset demonstrating high similarity to the original dataset (Fig S1). The combined dataset (*N* = 10,000) was used for training and internal validation with a split-sampling conducted in the ratio 3:1 to partition the development data into training and test sets [21].

### Temporal validation cohort

The EFGH study set out to establish incidence and consequences of *Shigella* medically attended diarrhea (MAD) within 7 country sites in Africa, Asia, and Latin America using cross-sectional and longitudinal study designs. MAD cases defined as children aged 6–35 months presenting with diarrhea (defined as ≥ 3 looser-than-normal stools within 24 h) that began within the past 7 days after ≥ 2 diarrhea-free days were enrolled from SHCs in the study catchment area [17]. Additional eligibility criteria included: residing within the pre-defined study catchment area; primary caregiver and child plan to remain at their current residence for at least the next 4 months; legal guardian consenting to child’s participation in the study as well willingness to be followed-up for 3 months post-enrolment; child is not being referred to a non-EFGH facility at the time of screening; and site enrollment cap has not been met. Follow-ups were conducted at week-4 (24–67 days) and month-3 (84–127 days). Our study utilized data from children enrolled in Kenya between 01 August, 2022 and 31 July, 2023 to temporally validate the champion model.

Information on demographic, socio-demographic, epidemiological and clinical characteristics were collected at enrollment by study personnel in both studies [18].

### Target variable

Consistent with previous studies [12, 13], we defined the target variable, LGF, as decrease of 0.5 HAZ or more (Δ HAZ ≥ − 0.5) within 49–91 days of enrollment in VIDA, or within 84–127 days in EFGH. We also computed change in HAZ per month of follow-up and categorized a negative change as LGF in our sensitivity analysis, similar to the definition used by Nasrin et al. [22]. We excluded children with implausible HAZ values (HAZ > 6 or < − 6 and change in (Δ) HAZ > 3; or length values that were > 1.5 cm lower at follow-up than at enrollment.

### Predictive variables and feature selection

A total of 68 potential candidate predictors collected at enrollment during both studies were considered, including demographic, household-level characteristics, illness history, anthropometric and clinical characteristics collected at enrolment. Missingness patterns were assessed among the features and the missing data points imputed using the Multiple Imputation by Chained Equations (MICE) package [23]. Furthermore, we conducted feature selection to reduce dimensionality, optimize performance, reduce computational complexity and enhance model interpretability. The feature selection was implemented using the Boruta package [24] an all relevant feature selection wrapper around the random forest algorithm that selects relevant features by comparing original attributes’ importance (contribution of each variable to the model’s predictive accuracy) with importance achievable at random using their permuted copies. Features that were either confirmed or tentative from the feature selection process were then used in model development. Moreover, among the confirmed and tentative features, we excluded variables that were not collected in both studies (breastfeeding).

### Statistical analysis

We compared patient characteristics of children with LGF versus those without. Proportions were reported for categorical variables and either chi-square or Fisher`s exact test were performed as appropriate. Wilcoxon rank sum tests were used to compare continuous variables as appropriate. We also compared the prevalence of LGF between the 2 studies.

### Model development and internal validation

To derive the LGF prediction model, we utilized 7 ML algorithms including: Random Forest (RF), Gradient Boosting (GBM), Naive Bayes (NB), Logistic regression (LR), Support vector machine (SVM), K-nearest neighbors (KNN) and Artificial Neural Networks (ANN). The predictive models were developed in the training dataset using 10-fold cross-validation [25], a valuable step in model development helping to obviate under-fitting or overfitting of the model and ensure robust and well-performing models. Due to the moderate class imbalance in our target variable (LGF), we employed sub-sampling techniques (over-sampling) within the resampling procedure to mitigate the negative impact of class disparity on model fitting [26]. We then conducted internal validation of the models on the test data evaluating performance using the following metrics: sensitivity, specificity, positive predictive value (PPV), negative predictive value (NPV) and F1-score. Receiver operating characteristic (ROC) curves were constructed and the area under the curve (AUC) and the precision-recall area under the curve (PRAUC) for each model were computed using the precrec package [27]. The ROC AUC is a threshold-independent metric that summarizes a model’s overall performance in discriminating between two classes. It represents the area under the ROC curve, which plots the true positive rate (sensitivity) against the false positive rate (1-specificity) at various classification thresholds. The PRAUC is a threshold-independent metric, particularly well-suited for imbalanced datasets. It summarizes model performance by capturing the area under the precision-recall curve, which illustrates the tradeoff between precision (the proportion of true positives among predicted positives) and recall (the proportion of true positives among all actual positives) across various thresholds. We determined the champion model as the model with the best AUC. We also assessed calibration in the developed models using Brier scores (the mean squared error between the actual outcome and the estimated probabilities), Spiegelhalter’s *z*-test (a formal measurement that serves as a proxy for calibration calculated from the decomposition of Brier score) and its accompanying p-value [28]. We used Platt scaling approach, in which model estimates are transformed by passing the estimates through a trained sigmoid function, to calibrate the champion model [28]. To enhance model interpretability, trust and fairness, we conducted explanatory model analysis (EMA) for the top two models using a model agnostic procedure to estimate SHapley Additive exPlanations (SHAPs) attributions, showing the magnitude and direction of association, drawing on the DALEX package [29].

### Temporal Validation and Business Value Evaluation

We further conducted temporal validation on the champion model to assess the robustness and generalizability of the model’s performance over time [30]. To evaluate the business value of the predictive model, modelplotr package [31] was used to build valuable evaluation plots (cumulative gains, cumulative lift, response and cumulative response plots). Descriptive analysis, predictive modelling for LGF and plotting were all performed in R version 4.2.2 [32].

## Results

**Fig. 1:**
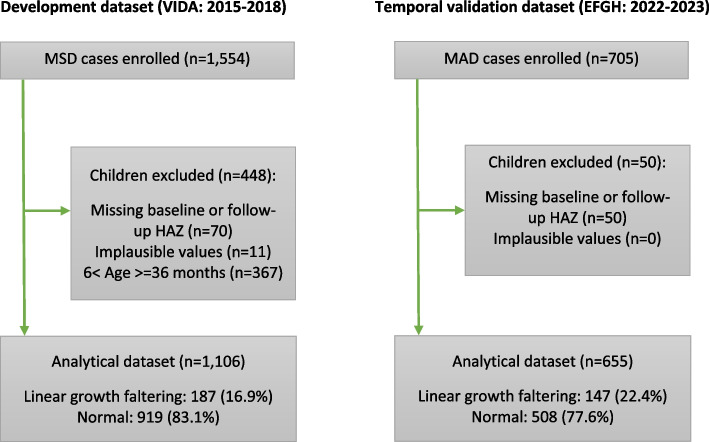
Flowchart of development and temporal validation studies conducted in Siaya County, Kenya. VIDA- Vaccine Impact on Diarrhea in Africa Study. EFGH-Enterics for Global Health Shigella Surveillance study. MSD-Moderate-to-Severe Diarrhea; MAD-Medically Attended Diarrhea

A total of 1,554 and 706 children were enrolled in the development and temporal validation cohorts, respectively. Among children aged 6–35 months enrolled, 1,106 (71.2%) and 655 (92.7%) had HAZ data that were plausible, respectively. Among those that had plausible HAZ data, 187 (16.9%) and 147 (22.4%) had LGF in the development and temporal validation cohorts, respectively (Fig. 1).

The median [interquartile range] ΔHAZ between enrollment and follow-up was − 0.21 [−0.42- −0.01] and − 0.24 [−0.48- −0.02] in the development and temporal validation cohorts, respectively. In the sensitivity analysis using the cut-off of negative change in HAZ, the prevalence of LGF was 1,051 (28.7%). Additionally, the constructed synthetic dataset had 8,527 observations and it closely replicated the propensity score distribution of the original development data (VIDA) as evidenced by the comprehensive descriptive analysis that compared each variable (Table S1).

The characteristics of VIDA participants at enrolment stratified by LGF status are shown in Table 1. Children who had LGF were younger than those who did not (Median age in months [IQR]: 11 [8–14] vs. 17 [11–24], *p* **<** 0.001). Furthermore, compared with those who did not have LGF, those with LGF had a higher respiratory rate (Median [IQR]: 38.5 [34.0–42.5] vs. 36.0[31.5–39.5], *p* < 0.001), a higher temperature (Median [IQR]: 37.1 [36.6–37.8] vs. 36.8 [36.4–37.5], *p* < 0.001) and more severe disease (Median Vesikari score [IQR]: 11 [9–12] vs. 10 [8–12], *p* < 0.001). Additionally, caretaker education, vomiting, wrinkled skin, restless, admission, and intravenous rehydration were significantly associated with LGF (Table 1).

**Table 1:** Characteristics of children aged < 5 years seeking care for moderate-to-severe diarrhea in Kenya stratified by Linear Growth faltering Status, 2015–2018

	Linear Growth Faltering		
Characteristics	Yes (*n* = 187)	No (*n* = 919)	*p*-value^*^
	*n* (%)	*n* (%)	
**Demograhic**			
Median age [IQR]	11 [8–14]	17 [11–24]	**< 0.001**
Age Category			
0–11 months	104 (55.6)	259 (28.2)	**< 0.001**
12–23 months	74 (39.6)	428 (46.6)	
24–59 months	9 (4.8)	232 (25.2)	
Gender: Female	83 (44.4)	428 (46.6)	0.584
**Household Details**			
Caretaker education ( > = Secondary )	78 (41.7)	305 (33.2)	**0.026**
<= 2 children under 5 yrs	167 (89.3)	839 (91.2)	0.387
<= 4 people sleeping	77 (41.2)	400 (43.6)	0.546
<= 3 Total Assets	158 (84.5)	812 (88.4)	0.142
Refined/Electric Primary Fuel Source^β^	5 (2.7)	39 (4.3)	0.313
Animal ownership	176 (94.1)	836 (91.0)	0.159
Improved water			
Safely managed	83 (44.3)	431 (46.9)	0.15
Basic	14 (7.5)	112 (12.2)	
Limited	28 (15.0)	125 (13.6)	
unimproved/Surface water	62 (33.2)	251 (27.3)	
Improved Sanitation			
Safely Managed and Basic	20 (10.7)	106 (11.5)	0.392
Limited	72 (38.5)	306 (33.3)	
Unimproved/Open Defecation	95 (50.8)	507 (55.2)	
**Clinical characteristics**			
**Reported by caretaker**			
Breastfeeding before diarrhea onset			
None	25 (13.4)	248 (37.9)	**< 0.001**
Exclusive	3 (1.6)	8 (0.9)	
Partial	159 (85.0)	563 (61.2)	
Median diarrhea days [IQR]	3 [2–3]	3 [2–4]	0.7196
Stool Type			
Simple watery	113 (60.4)	532 (57.9)	0.41
Rice watery	5 (2.7)	12 (1.3)	
Sticky/Mucoid	65 (34.8)	347 (37.8)	
Bloody	4 (2.1)	28 (3.1)	
Stool Count			
3	27 (14.4)	165 (18.0)	0.489
4–5	101 (54.0)	506 (55.0)	
6–10	55 (29.4)	228 (24.8)	
> 10	4 (2.1)	20 (2.2)	
Blood in stool	15 (8.0)	108 (11.8)	0.138
Vomiting	127(67.9)	531 (57.8)	**0.01**
Very Thirsty	156 (83.9)	752 (82.2)	0.582
Drinks poorly	47 (25.1)	232 (25.3)	0.962
Unable to drink	2 (1.1)	27 (2.9)	0.145
Belly Pain	109 (61.2)	508 (57.9)	0.404
Fever	142 (75.9)	709 (77.2)	0.720
Restless	151 (80.8)	710 (77.3)	0.295
Lethargy	123 (65.8)	600 (65.3)	0.898
unconscious	7 (3.7)	32 (3.5)	0.864
Rectal straining	55 (29.4)	211 (23.1)	0.066
Rectal prolapse	2 (1.1)	15 (1.6)	0.565
Cough	103 (55.1)	482 (52.5)	0.511
Difficulty breathing	32 (17.1)	124 (13.5)	0.197
Convulsion	3 (1.6)	17 (1.9)	0.818
**Currently**			
Very Thirsty	145 (78.4)	653 (71.7)	0.062
Drinks poorly	40 (21.5)	188 (20.5)	0.747
Sunken Eyes	171 (91.4)	792 (86.3)	0.054
Wrinkled skin	56 (30.6)	211 (23.0)	**0.029**
Restless	134 (71.7)	557 (60.6)	**0.004**
Lethargy/unconscious	23 (12.3)	151 (16.4)	0.157
Dry mouth	142 (75.9)	658 (71.7)	0.235
Fast breathing	24 (12.8)	100 (10.9)	0.44
Home ORS use	21 (11.2)	86 (9.4)	0.43
Home Zinc use	8 (4.3)	34 (3.7)	0.706
**Assessed by Clinician**			
Temperature [IQR]	37.1 [36.6–37.9]	36.8 [36.4–37.5]	**< 0.001**
Measured Fever (≥ 37.5^o^C)	99 (52.9)	342 (37.2)	**< 0.001**
Median Respiratory rate [IQR]	38.5 [34.0–42.5]	36.0 [31.5–39.5]	**< 0.001**
Chest indrawing	4 (2.1)	9 (1.0)	0.180
Sunken eyes	177 (94.7)	848 (92.3)	0.255
Dry mouth	183 (97.9)	903 (98.3)	0.71
Skin turgor (slow/very slow)	78 (41.7)	391 (42.6)	0.833
Mental Status			
Normal	73 (39.0)	380 (41.4)	0.052
Restless/Irritable	108 (57.8)	530 (57.7)	
Lethargic/Unconscious	6 (3.2)	9 (0.9)	
Rectal prolapse	0 (0)	3 (0.3)	0.434
Bipedal edema	2 (1.1)	5 (0.5)	0.337
Abnormal hair	9 (4.8)	43 (4.7)	0.937
Under Nutrition	21 (11.2)	109 (11.9)	0.807
Flaky Skin	2 (1.1)	5 (0.5)	0.409
Severe Acute Malnutrition (SAM)	24 (12.8)	78 (8.5)	0.061
Wasting	13 (7.0)	39 (4.2)	0.111
Admission	27 (14.4)	87 (9.5)	**0.043**
Diarrhea Duration (≥ 7 days)	70 (37.4)	327 (35.6)	0.631
any_antibiotic	78 (41.7)	402 (43.7)	0.609
Rotavirus vaccination doses			
0	2 (1.1)	19 (2.4)	0.385
1	8 (4.6)	25 (3.1)	
2	166 (94.3)	764 (94.5)	
ORS at facility	186 (99.5)	914 (99.9)	0.311
Zinc at facility	183 (97.9)	887 (96.9)	0.494
IV rehydration	31 (16.6)	92 (10.1)	**0.01**
Dehydration			
None	8 (4.3)	35 (3.8)	0.747
Some	126 (67.4)	645 (70.2)	
Severe	53 (28.3)	239 (26.0)	
Vesikari Score			
Mild	13 (7.0)	71 (7.7)	0.088
Moderate	75 (40.1)	442 (48.1)	
Severe	99 (52.9)	406 (44.2)	
Median Vesikari score [IQR]	11 [9–12]	10 [8–12]	**0.0003**
**Diagnosis**			
Dysentery	10 (5.4)	58 (6.4)	0.605
Malaria	85 (45.5)	361 (39.5)	0.131
Pneumonia	12 (6.4)	37 (4.1)	0.152
Bacterial Infection	14 (7.5)	93 (10.2)	0.258
Malnutrition	15 (8.0)	68 (7.4)	0.784

^*^P-value computed using either chi-square or Fisher`s exact test were performed as appropriate for categorical variables and Wilcoxon rank sum tests were used to compare continuous variables

^β-^ Includes electricity, propane, butane, natural gas; SAM defined as WHZ <−3 or MUAC < 115 millimeters, or the presence of bilateral pitting edema; ORS-Oral rehydration solution

**Fig. 2:**
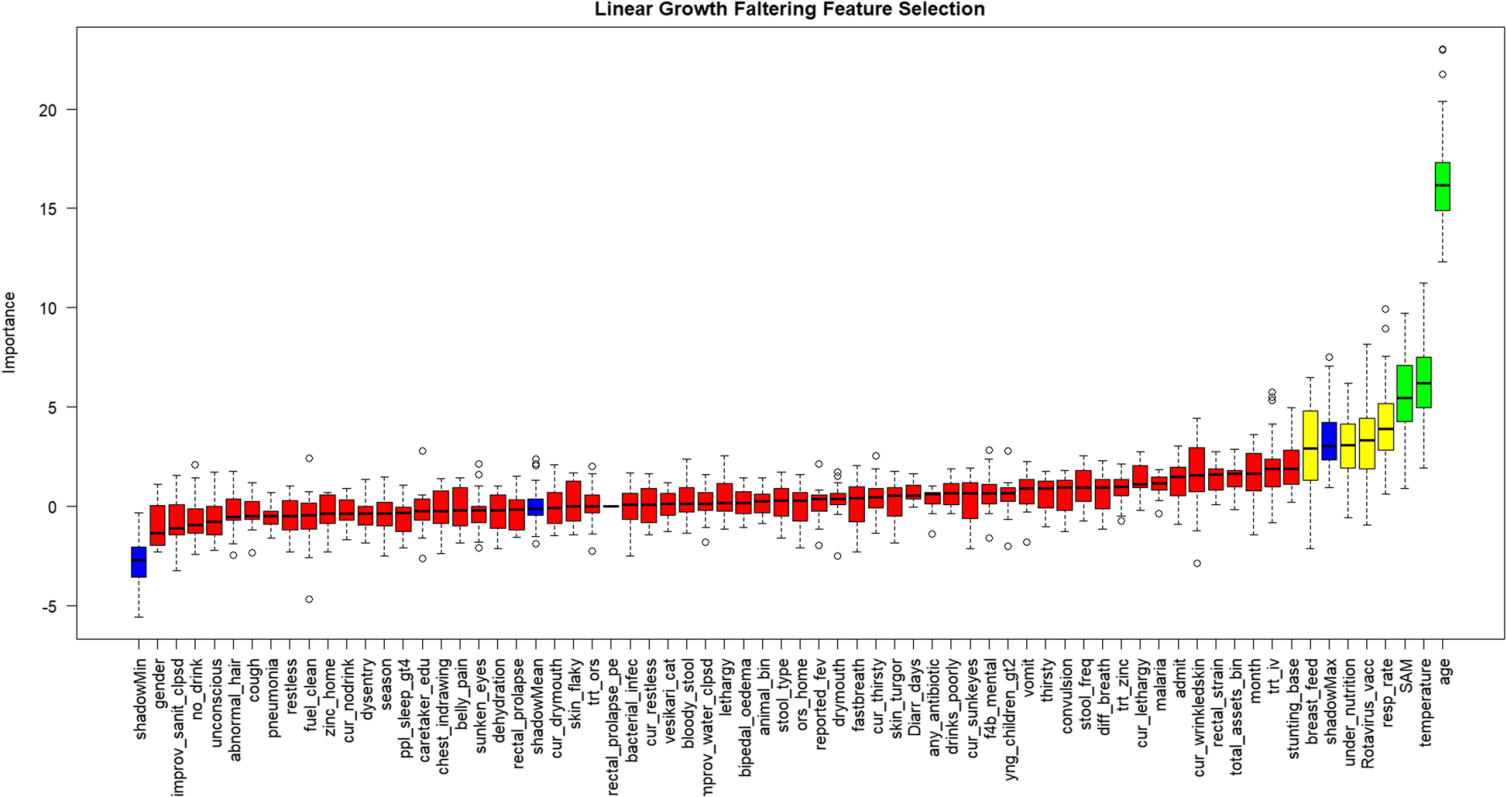
Feature selection for linear growth faltering among children aged < 5 years presenting with moderate to severe diarrhea in rural western Kenya, 2015-2018. Green, yellow, red and blue boxplots represent the Z scores of confirmed, tentative, rejected and shadow features, respectively. Confirmed and tentative features: *Age; temperature; respiratory rate; severe acute malnutrition (SAM); rotavirus vaccination; breastfeeding; skin turgor*

From the feature selection analysis, the confirmed variables in order of importance were age (16.6%), temperature (6.0%), and respiratory rate (4.1%) SAM (3.4%), rotavirus vaccination (3.3%), and skin turgor (2.1%) were tentative features (Fig. 2).

In addition to age, respiratory rate, and temperature, the following features were selected: confirmed (stunting at baseline [5.2%], vomit [4.0%], Vesikari score (3.7%) and sunken eyes [3.6%]) and tentative (bacterial infection diagnosis [2.5%]) in the sensitivity analysis using a cut-off of negative change in HAZ (Figure S2).

### Model performance

We evaluated seven ML algorithms in the prediction of LGF. From the developed models, sensitivity was highest in the RF model (80.7%), followed by the ANN (79.5%), SVM (77.3%), NB (76.5%), GBM (75.6%), LR (75.4%) and lowest in the KNN model (72.4%). The specificity ranged from 58.2 to 71.8%. Specifically, the specificity of the GBM model was the highest (71.8%), followed by RF (70.1%), LR (61.9%), NB and SVM (61.6%), KNN (61.4%) and lowest.

in the ANN model (58.2%). The PPV ranged between 27.4 − 34.9% while the NPV ranged between 92.3 − 94.8%. The AUC of the models ranged from 73.4 to 83.5% with the GBM model having the highest AUC (83.5%, 95% Confidence Interval [95% CI]: 81.6–85.4) (Table 2).

**Table 2:** Model performance of linear growth faltering prediction^β^ models using combined data (original and synthetic data)

Algorithm^*^	Sensitivity % [95% CI]	Specificity % [95% CI]	PPV % [95% CI]	NPV % [95% CI]	F1-Score [95% CI]	AUC % [95% CI]	PRAUC % [95% CI]
RF	80.7 [76.5–84.4]	70.1 [68.1–72.1]	34.9 [31.9–38.0]	94.8 [93.6–95.9]	48.7 [16.8–59.7]	82.8 [80.8–84.8]	96.0 [93.8–96.2]
GBM	75.6 [71.2–79.7]	71.8 [69.8–73.7]	34.7 [31.6–37.9]	93.7 [92.4–94.8]	47.6 [13.9–74.5]	83.5 [81.6–85.4]	96.2 [94.9–96.5]
NB	76.1 [71.7–80.1]	61.6 [59.5–63.7]	28.2 [25.6–31.0]	92.8 [91.4–94.1]	40.2 [12.0–42.4]	75.6 [73.3–77.9]	94.0 [92.1–95.0]
LR	75.4 [70.9–79.4]	61.9 [59.7–64.0]	28.2 [25.5–30.9]	92.7 [91.2–94.0]	38.2 [3.1–64.2]	73.7 [71.3–76.1]	93.0 [91.1–94.0]
SVM	77.3 [73.0–81.2]	61.6 [59.5–63.7]	28.6 [25.9–31.3]	93.2 [91.7–94.5]	41.7 [9.3–56.8]	73.4 [71.0–75.8]	93.0 [91.6–94.1]
KNN	72.4 [69.7–75.0]	61.4 [59.3–63.5]	27.6 [25.0–30.3]	92.3 [90.8–93.6]	40.2 [6.7–67.0]	74.8 [72.3–77.2]	93.0 [90.8–93.6]
ANN	79.5 [75.3–83.3]	58.2 [56.1–60.4]	27.4 [24.9–30.0]	93.5 [92.0–94.7]	40.8 [9.8–58.5]	73.6 [71.3–76.0]	93.0 [90.9–94.1]

*95% CI* 95% Confidence Interval, *PPV* Positive Predictive Value, *NPV* Negative Predictive Value, *AUC* Area under the Curve, *PRAUC* Precision Recall Area under the Curve

^*^RF-Random Forest; GBM-Gradient Boosting; NB- Naïve Bayes; LR-Logistic Regression; SVM- Support vector machine; KNN-K-nearest neighbors; ANN-Artificial Neural Networks

^β−^ Linear growth faltering defined as Δ HAZ ≥ − 0.5

The GBM model was chosen as the champion model. The receiver operating characteristic (ROC) curves for LGF prediction models are shown in Figure S3. Moreover, in the sensitivity analysis using only the VIDA data in development, the model performance ranged between 63.0 and 82.6%, 55.9–78.6%, 27.3–33.7%, 91.0–94.2%, 40.3–44.3%, 68.0–75.5%, and 90.6–94.4% for sensitivity, specificity, PPV, NPV, F1-score, AUC and PRAUC, respectively (Table 3). All models showed a decline in predictive performance during sensitivity analysis except for the SVM model, which had a marginal increase.

**Table 3:** Model performance of linear growth faltering prediction ^β^ models using original training data only

Algorithm^*^	Sensitivity % [95% CI]	Specificity % [95% CI]	PPV % [95% CI]	NPV % [95% CI]	F1-Score [95% CI]	AUC % [95% CI]	PRAUC % [95% CI]
RF	52.2 [36.9–67.1]	78.6 [72.7–83.7]	32.9 [22.3–44.9]	89.1 [84.0–93.0]	40.3 [10.9–51.4]	70.3 [61.8–78.7]	90.6 [88.4–90.8]
GBM	80.4 [66.1–90.6]	63.3 [56.7–69.6]	30.6 [22.5–39.6]	94.2 [89.2–97.3]	44.3 [12.9–55.8]	75.5 [68.2–82.8]	93.6 [92.3–93.9]
NB	63.0 [47.5–76.8]	75.1 [69.0–80.6]	33.7 [23.9–44.7]	91.0 [86.0–94.7]	43.9 [5.6–60.3]	73.6 [66.1–81.2]	93.0 [91.1–94.0]
LR	73.9 [58.9–85.7]	63.3 [56.7–69.6]	28.8 [20.8–37.9]	92.4 [87.0–96.0]	41.5 [7.8–52.1]	73.8 [67.0–80.5]	93.9 [92.0–94.9]
SVM	71.7 [56.5–84.0]	65.9 [59.4–72.1]	29.7 [21.4–39.1]	92.1 [86.8–95.7]	42.0 [7.2–51.9]	75.2 [68.9–81.5]	94.4 [93.0–95.5]
KNN	82.6 [68.6–92.2]	56.8 [50.1–63.3]	27.7 [20.4–36.0]	94.2 [88.9–97.5]	41.5 [12.2–51.8]	73.1 [66.3–79.9]	93.6 [91.4–94.2]
ANN	82.6 [68.6–92.2]	55.9 [49.2–62.4]	27.3 [20.1–35.5]	94.1 [88.7–97.4]	41.1 [12.0–57.9]	68.0 [60.5–75.6]	91.4 [89.3–92.5]

*95% CI* 95% Confidence Interval, *PPV* Positive Predictive Value, *NPV* Negative Predictive Value, *AUC* Area under the Curve, *PRAUC* Precision Recall Area under the Curve

^*^RF Random Forest; GBM-Gradient Boosting; NB- Naïve Bayes; LR-Logistic Regression; SVM- Support vector machine; KNN-K-nearest neighbors; ANN-Artificial Neural Networks

^β−^ Linear growth faltering defined as Δ HAZ ≥ − 0.5

In the sensitivity analysis using the second definition of LGF (negative change in HAZ), the model performance ranged between 45.8 and 73.1%, 53.2–76.6%, 79.0–90.5%, 28.6–48.5%, 58.3–80.9%, 58.0–82.4%, and 29.0–62.6% for sensitivity, specificity, PPV, NPV, F1-score, AUC and PRAUC, respectively (Table S2). In this scenario, all models exhibited a drop in predictive performance except for the SVM model, which had a marginal increase and the RF model which registered same performance as in the primary analysis.

Overall the Brier scores were relatively high and ranged between 0.19 and 2.50 (Table 4).The Spiegelhalter’s p-value showed that all the models were not properly calibrated (*p* < 0.05). The performance of the calibrated GBM model was largely similar to its uncalibrated form with the model having an AUC of 83.7%.

**Table 4:** Calibration results of linear growth faltering prediction models

Algorithm^*^	Brier Score	Spiegelhalter Z-score	Spiegelhalter *p*-value
RF	0.19	16.83	< 0.0001
GBM	2.50	208.10	< 0.0001
NB	2.18	101.02	< 0.0001
LR	2.16	85.02	< 0.0001
SVM	2.16	85.88	< 0.0001
KNN	2.21	109.88	< 0.0001
ANN	2.17	84.07	< 0.0001

^*^RF-Random Forest; GBM-Gradient Boosting; NB- Naïve Bayes; LR-Logistic Regression; SVM- Support vector machine; KNN-K-nearest neighbors; ANN-Artificial Neural Networks

### Explanatory model analysis

**Fig. 3:**
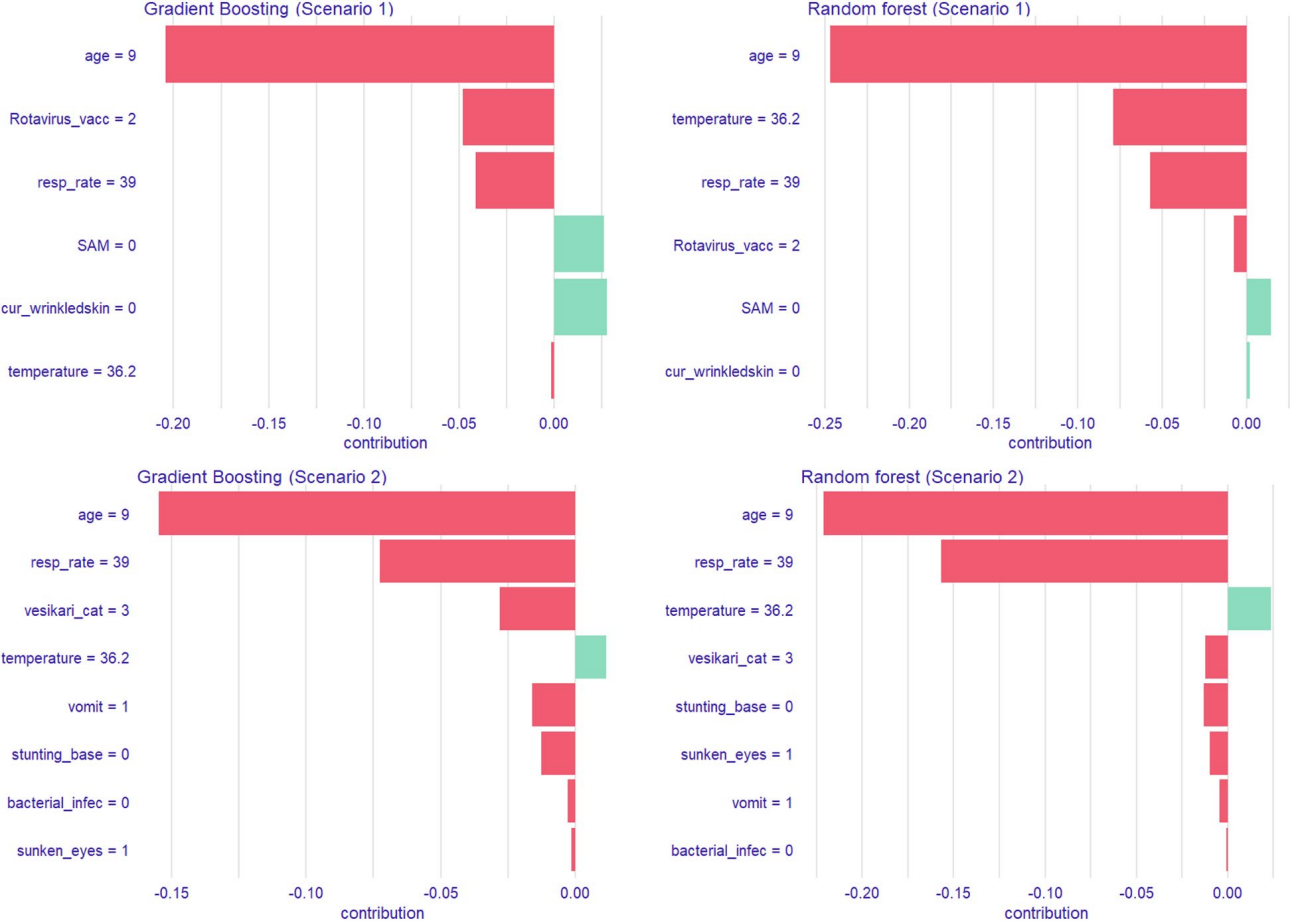
SHAP attributions for the Top 2 linear growth faltering models. *Scenario 1- Predicting linear growth faltering using a cut-off of Δ HAZ ≥ − 0.5. * age=9: 9 months; Rotavirus_vacc =2:2 doses of rotavirus vaccine; cur_wrinkledskin=0: normal skin; SAM=0: No severe acute malnutrition (SAM). *Scenario 2- Predicting linear growth faltering using change in haz/month (negative change in linear growth is deemed growth faltering). *age=9: 9 months; vesikari_cat=3: Severe disease based on Vesikari score; vomit=1: Vomitting; Stunting_base=0: No stunting at baseline; bacterial_infec=0: No bacterial infection; sunken_eyes =1: sunken eyes

The EMA results for the top 2 models in the primary analysis were similar though the degree of importance varied across models with no SAM, no skin turgor, no rotavirus vaccine, age, elevated temperature and respiratory rate being predictive of LGF (Fig. 3). Similarly, in the sensitivity analysis using the second definition of LGF, the direction of association was similar between the two models although the magnitude of importance varied. In addition to age, respiratory rate and temperature, the following factors were also identified to be predictive of LGF: severity of disease, no vomiting, stunting at baseline, bacterial infection and lack of sunken eyes (Fig. 3).

### Business value evaluation of Champion Model

**Fig. 4:**
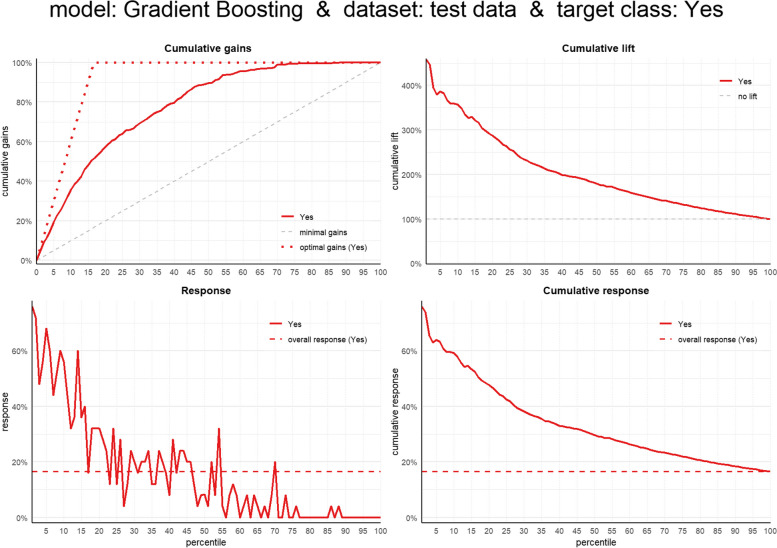
Business value plots for the Gradient Boosting (GBM) Model for linear growth faltering

From the business value evaluation of our champion model (GBM), the cumulative gains plot shows that the model is able to select ~ 60% of the target class (LGF) if we select the top-20% cases based on our model. Additionally, from the cumulative lift plot, our champion model is able to identify ~ 3 times higher number of the target class compared to a random selection if we pick the top-20% observations based on model probability. Lastly, from the cumulative response plot, 48% of observations in the top-20% cases based on model probability belong to the target class (Fig. 4).

### Temporal validation

**Fig. 5:**
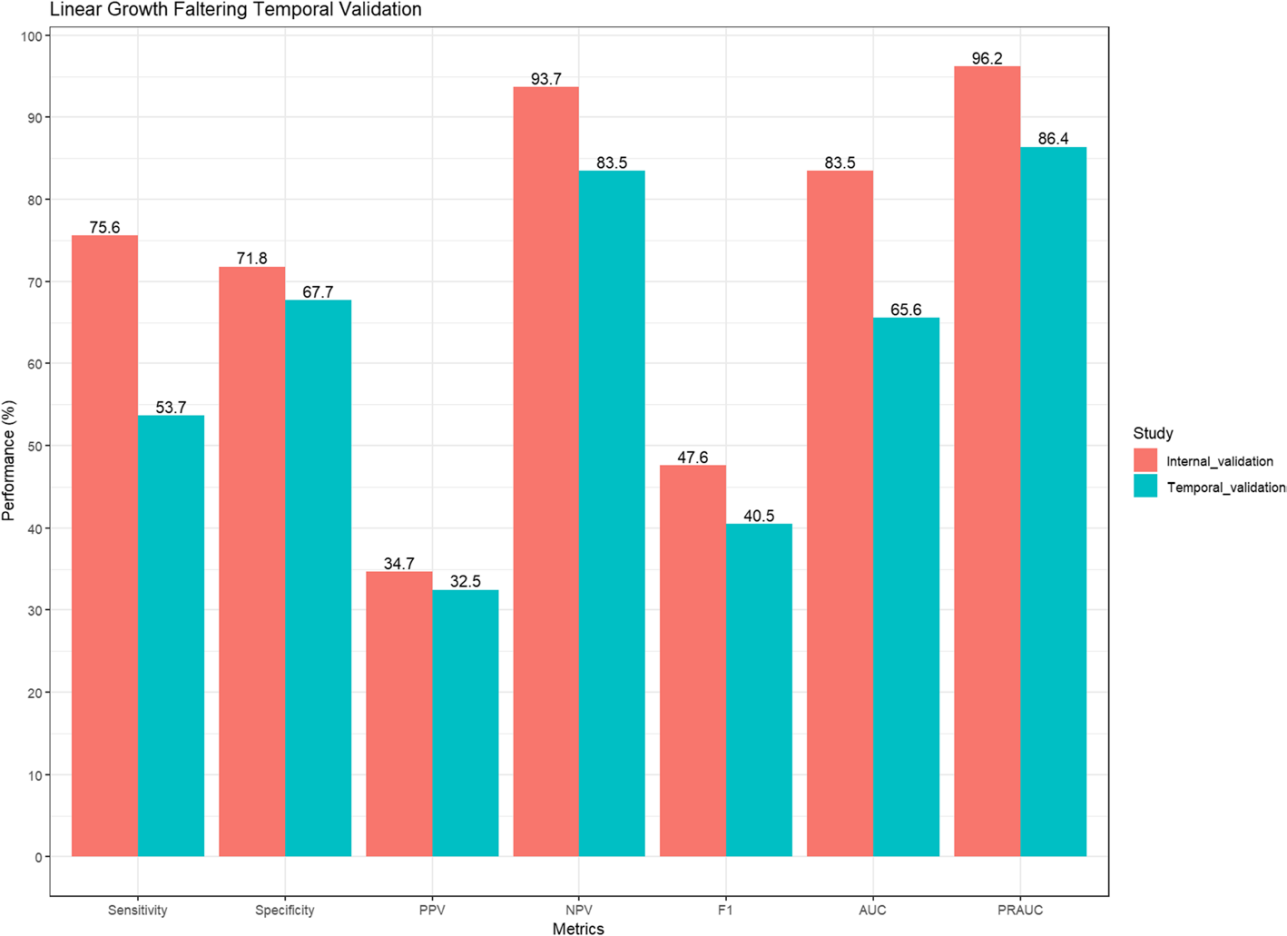
Performance of champion model in development (2015-2018) and temporal validation (2022-2023) datasets. PPV- Positive Predictive Value; NPV- Negative Predictive Value; AUC- Area under the Curve; PRAUC- Precision Recall Area under the Curve

We observed a decline in model performance on the temporal validation dataset with the AUC dropping by ~ 18%. Additionally, all metrics dropped in temporal validation with the GBM model achieving 53.7%, 67.7%, 32.5%, 83.5%, 40.5%, 65.6% and 86.4% for sensitivity, specificity, PPV, NPV, F1-score, AUC and PRAUC, respectively (Fig. 5).

## Discussion

The study findings illuminate a comprehensive exploration into the prediction of LGF among pediatric patients presenting with diarrhea, employing a robust ML framework. The study involved the development and temporal validation of predictive models using diverse cohorts, revealing distinct prevalence rates and influencing factors associated with LGF. Key features linked to this outcome, such as age, rotavirus vaccination, respiratory rate, temperature and SAM, were identified through extensive feature selection and their impact on risk prediction was estimated using SHAP attribution. The ML algorithms exhibited varying performance with GBM model emerging as the champion model, demonstrating promising business value. However, the temporal validation uncovered a notable decline in model performance, emphasizing the dynamic nature of health data and the need for ongoing model evaluation and adaptation.

Despite the impact of rotavirus vaccine introduction on the epidemiology of diarrhea and pathogen landscape, we identified similar predictors, in addition to rotavirus vaccination, to previous modelling efforts [12, 13] that used data collected pre-vaccine introduction─ age,, respiratory rate, temperature, absence SAM and stunting at baseline. This finding underscores the enduring importance of these risk factors and the need for comprehensive, sustained, and adaptable public health strategies to combat LGF. Furthermore, we observed that rotavirus vaccination was inversely associated with LGF a finding that is consistent with those of Loli and Carcamo who studied the impact of vaccination on HAZ in Peruvian children aged 6–60 months [33]. This finding could be due to rotavirus vaccination substantially reducing the incidence and severity of rotavirus infections, curbing the immediate impact of diarrheal diseases on nutrient absorption and consequently diarrhea-mediated growth faltering [33]. Bolstering rotavirus vaccination is a possible strategy that could be leveraged by policy makers and public health experts to reduce stunting in such settings. Moreover, from a modelling perspective, this finding on predictors generates confidence in the relevance and stability of these variables in different contexts and epidemiological periods, enhancing model transferability and generalizability.

These variables have been documented as risk factors for LGF in previous studies. Specifically, age is a significant determinant of LGF among pediatric populations following a diarrheal episode [12, 13, 34]. Infants and very young children face heightened vulnerability to nutritional and health challenges due to their ongoing physiological maturation, which is exacerbated during diarrheal illness leading to pronounced impacts on nutrient absorption and utilization cumulatively contributing to a heightened prevalence of LGF among younger children. Stunting has been shown to be irreversible to a large extent after reaching 24 months of age [35]. Therefore, the timely identification of at-risk children (infants and toddlers) facilitates the implementation of effective preventive strategies during this critical window of opportunity in early childhood. Contrary to existing evidence [36, 37], we observed children without SAM to be at increased risk of LGF. Despite majority of factors predisposing children to SAM and stunting being similar, we observed a discordant relationship between the two and this may require further investigation to gain insights into this finding. Elevated baseline temperature and respiratory rate signal are markers of disease severity, and particularly those affecting the gastrointestinal tract, may lead to nutritional deficiencies and hinder linear growth [12, 13]. Additionally, elevated respiratory rate and temperature may indicate increased energy expenditure, potentially due to the body’s efforts to combat infections or inflammation. This increased energy demand can divert resources away from growth-related processes, impacting linear growth.

Tree-based ensembles showed good predictive performance with the GBM model narrowly outperforming the RF model in the prediction of LGF. Our champion model outperformed existing models by Brander et al. (AUC = 67.0%) [12] and (AUC = 75.0%) Ahmed et al. [13]. The improvement in model performance could be attributed to the robust modelling approach employed. Moreover, the predictive prowess of tree-based ensembles may have also contributed to this improvement. This strong discriminatory ability of the champion model has significant public health implications as it reinforces the feasibility and efficacy of ML algorithms in timely identification of children, at increased risk of LGF, for early nutritional and healthcare interventions. The model can enhance the efficiency of resource allocation by facilitating targeted screening as well as providing healthcare providers with a valuable tool for informed decision-making, enabling tailored interventions based on individual children risk profiles. The champion model could be implemented as web-based application using platforms like R-shiny or Plumber [38, 39], or integrated directly into electronic health record systems [40] to ensure alignment with clinical workflows. Deploying the model through these straightforward and adaptable methods would enable quick adoption in clinical settings, supporting clinicians in promptly identifying at-risk patients and enhancing clinical decision-making. However, the decline in model performance during temporal validation while consistent with findings from Ahmed et al. [13] raises important considerations. Spectral differences in the severity of diarrhea among children in the development and validation cohorts, coupled with potential shifts in the study population over time, highlight challenges in maintaining consistent predictive accuracy. This finding highlights the need for monitoring and periodic retraining of the model in order to maintain its predictive performance.

Our primary analysis that used combined data (VIDA and synthetic data) in model development had better performance than the sensitivity analysis that only used VIDA data. This result emphasizes the importance of synthetic data in addressing challenges associated with imbalanced, limited, or privacy-sensitive real-world datasets, providing a means to augment and diversify the data pool [41, 42]. This approach overcomes issues of data scarcity, facilitates more comprehensive model training, and enhances generalization. It contributes to overcoming biases, ensuring model fairness, and accommodating the complexity of risk factors influencing a health outcome. Ultimately, the strategic use of synthetic data strengthens the reliability, generalizability, and ethical integrity of predictive models, offering a pathway for more effective and personalized healthcare interventions. However, synthetic data may advance bias propagation since any biases in the primary data will be reflected in the generated data and this may perpetuate and even exacerbate healthcare disparities if they exist [43]. In addition to the quality of synthetic data being largely dependent on the underlying primary data, synthetic datasets may fail to encompass the complete range of variations and intricacies found in real-world data. Furthermore, in the second sensitivity analysis using a cutoff of any negative change in HAZ, we observed a substantial decline in model performance compared to using a cutoff of a decrease of 0.5 HAZ or more. These results imply that using a specific cutoff criteria for defining LGF can significantly impact the performance of the predictive model. Different cutoff criteria may be more appropriate in different contexts, and the choice should be informed by clinical expertise and relevance considering the specific context of the healthcare setting, study population (varying age categories), and the clinical significance of HAZ changes. It also underscores the dynamic nature of model performance, necessitating ongoing evaluation and adaptation to maintain optimal cutoff criteria.

Our study, while commendable, has limitations, notably the exclusion of pathogen data during model development to maintain practical applicability, despite its influence on LGF. Future research should address this gap, as well as focus on the acceptability and impact of ML models on clinical practice and patient outcomes. The cost-effectiveness of deploying these models is also crucial for practical implementation in diverse healthcare settings. Exploring these facets will contribute significantly to enhancing understanding and ensuring the effective use of ML models in healthcare.

## Conclusion

The study’s findings emphasize the enduring relevance of established predictors of LGF. Addressing multifaceted challenges in pediatric LGF requires sustained efforts with adaptive interventions for these risk factors. The study demonstrates the practical use of ML algorithms for rapid identification of at-risk children for early nutritional and healthcare interventions. The model can enhance the efficiency of resource allocation by facilitating targeted screening as well as providing healthcare providers with a valuable tool for informed decision-making, enabling tailored interventions based on individual children risk profiles. However, a decline in model performance during temporal validation highlights the dynamic nature of health data, necessitating continuous evaluation and adaptation. Additionally, the study shows the viability of integrating synthetic data to enhance model robustness, providing a pathway for more comprehensive and ethical predictive modeling in healthcare.

## Supplementary Information

Supplementary Material 1.

## Data Availability

The data used for the modelling in this study belongs to KEMRI and restrictions apply to the availability of these data.
