# Supplementary material for: Predictive modelling of linear growth faltering among pediatric patients with Diarrhea in Rural Western Kenya: an explainable machine learning approach
Source: BMC Med Inform Decis Mak. 2024 Dec 2;24:368. doi: 10.1186/s12911-024-02779-7 (PMC11613762; doi:10.1186/s12911-024-02779-7)
Supplement: Supplementary file 1 — Supplementary Material 1. [file 12911_2024_2779_MOESM1_ESM.pdf]

Supplemental Materials

Figure S1. Histogram comparison for each predictor variable comparing the aggregate demographic characteristics of the original training dataset (n = 1,473) against synthetic dataset (n = 8,527).

Figure S2. Feature selection for linear growth faltering (delta_haz/month) among children aged < 5 years presenting with moderate to severe diarrhea in rural western Kenya, 2015-2018

Figure S3. Receiver operating characteristic curve for linear growth faltering prediction models

Table S1. Statistical analysis comparing synthetic data tables to the original training dataset (n = 8,527)

Table S2. Model performance of line

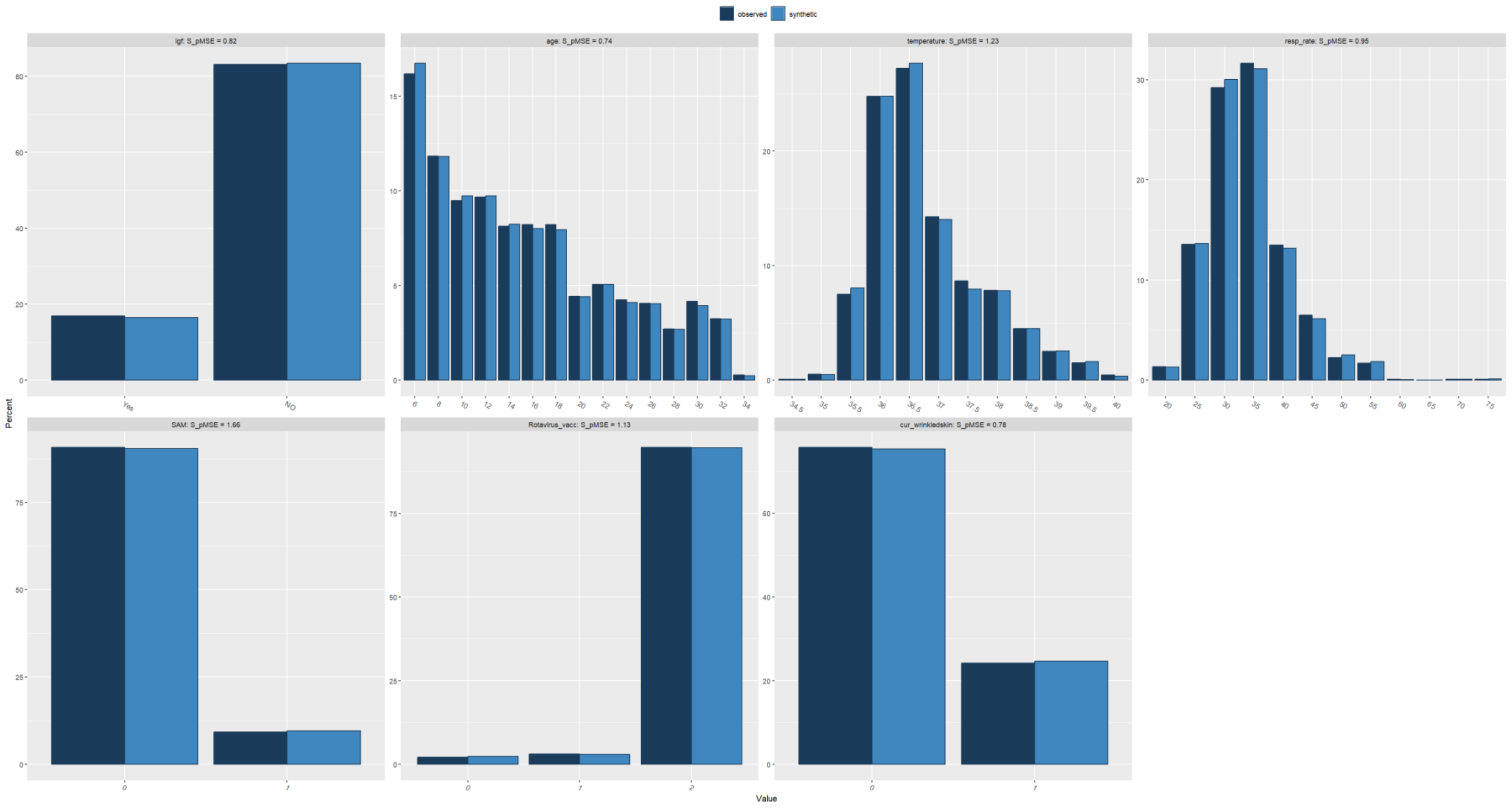

Figure S1. Histogram comparison for each predictor variable comparing the aggregate demographic characteristics of the original training dataset (n = 1,473) against synthetic dataset (n = 8,894).

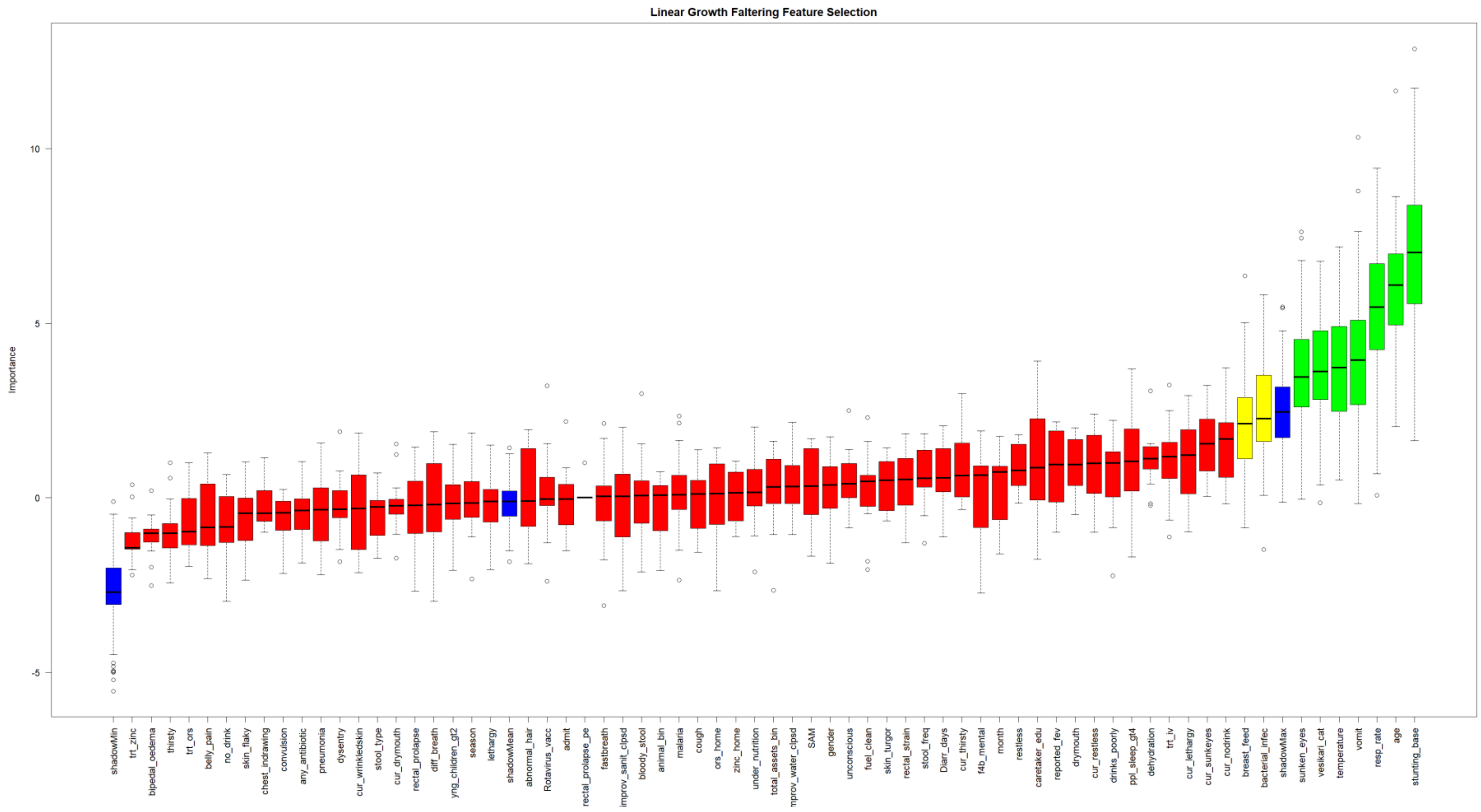

Green, yellow, red and blue boxplots represent the Z scores of confirmed, tentative, rejected and shadow features, respectively.
Confirmed and tentative features: *stunting at baseline;age; respiratory rate; vomit;temperature; vesikari_cat; sunken eyes; bacterial infection; breastfeeding.*

Figure S2. Feature selection for linear growth faltering (delta_haz/month) among children aged < 5 years presenting with moderate to severe diarrhea in rural western Kenya, 2015-2018

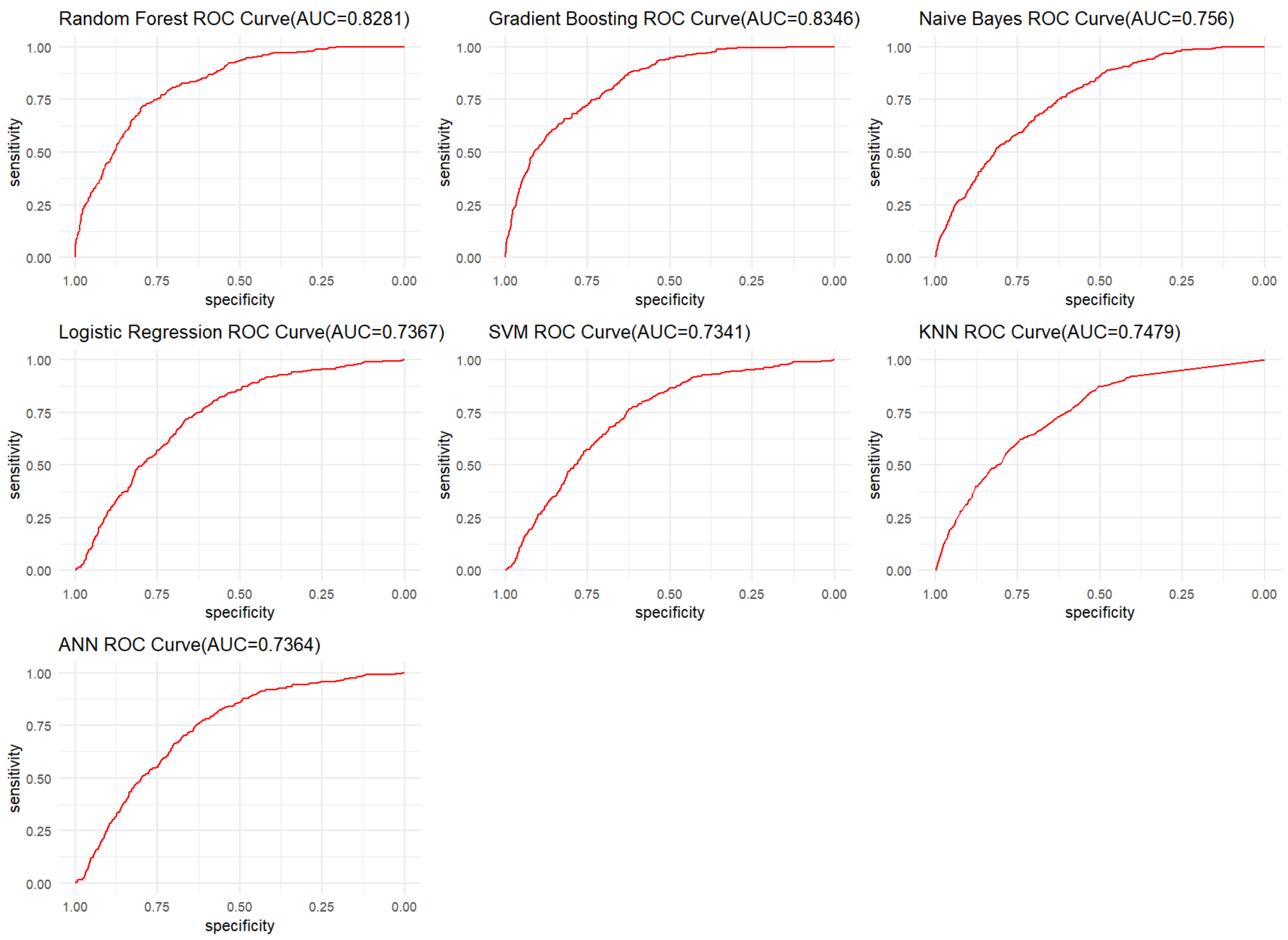

*RF-Random Forest; GBM-Gradient Boosting; NB- Naïve Bayes; LR-Logistic Regression; SVM- Support vector machine; KNN-K-nearest neighbors; ANN-Artificial Neural Networks; ROC-Receiver operating characteristic curve; AUC-Area under the curve

Figure S3. Receiver operating characteristic curve for linear growth faltering prediction models

Table S1. Statistical analysis comparing synthetic data tables to the original training dataset (n = 8,894)

	**Synthetic Data**	
	**n=8,894**	
**Variables**	**pMSE**	**S_pMSE**
lgf	0.0000010	0.820503
age	0.0000030	0.743468
temperature	0.0000050	1.228149
resp_rate	0.0000040	0.947305
SAM	0.0000020	1.659631
Rotavirus_vacc	0.0000020	1.131343
cur_wrinkledskin	0.0000010	0.779434

pMSE- propensity score mean-squared-error; S_pMSE- standardized ration of propensity score mean-squared error.

Table S2. Model performance of linear growth faltering$^{\beta}$ prediction models using combined data

Algorithm	Sensitivity % [95% CI]	Specificity % [95% CI]	PPV % [95% CI]	NPV % [95% CI]	F1-Score [95% CI]	AUC % [95% CI]	PRAUC % [95% CI]
RF	73.1 [71.1-75.1]	76.6 [73.1-79.9]	90.5 [88.9-91.9]	48.5 [45.3-51.6]	80.9 [65.2-97.0]	82.4 [80.5-84.2]	62.6 [59.4-65.8]
GBM	71.2 [69.0-73.2]	72.4 [68.7-75.9]	88.7 [87.0-90.2]	45.3 [42.2-48.5]	80.4 [76.6-96.4]	78.0 [76.0-80.1]	53.0 [50.2-55.5]
NB	66.0 [63.9-68.2]	53.2 [49.2-57.2]	81.1 [79.0-83.0]	34.1 [31.1-37.2]	72.8 [58.3-85.8]	62.2 [59.7-64.7]	34.3 [32.8-36.6]
LR	56.9 [54.7-59.2]	54.0 [50.0-58.0]	79.0 [76.7-81.1]	29.3 [26.7-32.0]	64.9 [50.1-89.9]	56.9 [54.3-59.5]	29.0 [26.5-31.9]
SVM	58.5 [56.3-60.8]	60.0 [56.0-63.9]	81.6 [79.4-83.6]	32.3 [29.6-35.1]	68.2 [62.2-94.3]	62.9 [60.4-65.4]	35.6 [32.3-39.0]
KNN	65.0 [62.8-67.1]	73.7 [70.1-77.1]	88.2 [86.4-89.9]	41.0 [38.1-43.9]	74.8 [59.9-97.0]	77.3 [75.2-79.4]	54.0 [51.2-57.3]
ANN	45.8 [43.6-48.1]	65.8 [61.9-69.5]	80.2 [77.7-82.6]	28.6 [26.3-31.0]	58.3 [43.6-85.4]	58.0 [55.5-60.6]	31.0 [29.1-32.8]

$^{\beta}$- Linear growth faltering defined as negative change in linear growth

*RF-Random Forest; GBM-Gradient Boosting; NB- Naïve Bayes; LR-Logistic Regression; SVM- Support vector machine; KNN-K-nearest neighbors; ANN-Artificial Neural Networks; 95% CI- 95% Confidence Interval; PPV- Positive Predictive Value; NPV- Negative Predictive Value; AUC- Area under the Curve; PRAUC- Precision Recall Area under the Curve
